# Human Amniotic Fluid Stem Cells Ameliorate Thioglycollate-Induced Peritonitis by Increasing Tregs in Mice

**DOI:** 10.3390/ijms23126433

**Published:** 2022-06-09

**Authors:** Yushi Abe, Daigo Ochiai, Masako Taguchi, Seiji Kanzaki, Satoru Ikenoue, Yoshifumi Kasuga, Mamoru Tanaka

**Affiliations:** 1 Department of Obstetrics & Gynecology, Keio University School of Medicine, Tokyo 160-8582, Japan; y.abe@keio.jp (Y.A.); masako1204@keio.jp (M.T.); skanzaki@stemcell.co.jp (S.K.); sikenoue.a3@keio.jp (S.I.); 17yoshi23.k@gmail.com (Y.K.); mtanaka@keio.jp (M.T.); 2 StemCell Institute Inc., Tokyo 105-0004, Japan

**Keywords:** amniotic fluid stem cells, mesenchymal stem cells, peritonitis, Treg

## Abstract

Mesenchymal stem cells (MSCs) affect immune cells and exert anti-inflammatory effects. Human amniotic fluid stem cells (hAFSCs), a type of MSCs, have a high therapeutic effect in animal models of inflammation-related diseases. hAFSCs can be easily isolated and cultured from amniotic fluid, which is considered a medical waste. Hence, amniotic fluid can be a source of cells for MSC therapy of inflammatory diseases. However, the effect of hAFSCs on acquired immunity in vivo, especially on regulatory T cells, has not yet been fully elucidated. Therefore, in this study, we aimed to understand the effects of hAFSCs on acquired immunity, particularly on regulatory T cells. We showed that hAFSCs ameliorated the thioglycollate-induced inflammation by forming aggregates with host immune cells, such as macrophages, T cells, and B cells in the peritoneal cavity. Further, the regulatory T cells increased in the peritoneal cavity. These results indicated that, in addition to helping the innate immunity, hAFSCs could also aid the acquired immune system in vivo against inflammation-related diseases by increasing regulatory T cells.

## 1. Introduction

Mesenchymal stem cells (MSCs) affect immune cells, exert a strong anti-inflammatory effect, and can be easily isolated and cultured from adipose tissue, bone marrow, and fetal tissues [1,2,3,4,5]. There are over 600 clinical trials listed at www.clinicaltrials.gov (accessed on 30 October 2021) utilizing MSCs; further, MSC-based drugs for graft-versus-host diseases are commercially available [6,7]. MSCs regulate immune responses and exert anti-inflammatory effects via the regulation of immune effector cells such as T, B, natural killer (NK) and dendritic cells, and macrophages [6,8,9]. Moreover, MSCs may be used therapeutically for other diseases without any effective treatment at present, including cerebral and myocardial infarction [9,10]. Among MSCs, human amniotic fluid stem cells (hAFSCs) are some of the most promising cells for MSC therapy. hAFSCs meet the MSC criteria and can be conveniently isolated and cultured from amniotic fluid [5]. Growth factors, chemokines, cytokines and hormones have been shown to induce the amplification of hAFSCs [11,12]. Amniotic fluid is obtained in large quantities as medical waste during amniocentesis, amnioreduction, delivery, and cesarean section. To date, hAFSCs have been shown to have anti-inflammatory effects on animal models of various diseases [13,14,15,16,17,18].

The anti-inflammatory effect of MSCs is critical for various biological processes [6,19]. T cells, B cells, NK cells, and macrophages are mainly involved in immunity caused by inflammation. The acquired immunity is activated in response to stimulation by foreign substances. It is characterized by high specificity and immune memory. In contrast, innate immunity recognizes foreign substances by targeting molecular patterns specific to microorganisms. Compared to acquired immunity, innate immunity is less specific and has no immune memory. However, it has the advantage of an immediate response to pathogen invasion [20]. MSCs, including hAFSCs, modulate both innate and acquired immunity, resulting in anti-inflammatory effects, but the mechanism underlying such effects has not been fully elucidated.

The relationship between hAFSCs and T cells, particularly Tregs, which play an important role in immune responses, requires investigation, as there have been fewer reports on the effects of hAFSCs on acquired immunity than those on the effects of hAFSCs on the macrophages in the innate immune system. In addition, in our previous study we attempted, unsuccessfully, to ascertain this relationship. In brief, we created a rat model of neonatal sepsis by administering LPS intraperitoneally and evaluated the therapeutic effects of hAFSCs. We also showed that the anti-inflammatory effect of hAFSCs resulted from the regulation of the inflammatory responses of macrophages with or without direct contact in the abdominal cavity [18,21]. However, it was concluded that neonatal immunity is predominantly innate, carried by macrophages, and that the model is not compatible with sufficiently investigating the acquired immune system.

We therefore focused on the peritonitis model, in which acquired immunity is known to be involved. Peritonitis is one of the most life-threatening inflammatory diseases [22,23]. Several rodent models have been developed to evaluate the pathophysiology of peritonitis and study the effects of various therapeutic interventions designed against the disease. Intraperitoneal administration of thioglycollate (TG) [24] or lipopolysaccharide (LPS) [25] and cecal ligation and puncture [26] are often used to develop rodent models of peritonitis [27]. The immune system of the abdominal cavity responds rapidly to bacteria that may be released by rupture of the intestinal tract, and both innate and acquired immunity are critical players in this response [28,29]. 

Several studies have explored the relationship between MSCs and acquired immunity using models of peritonitis. MSCs can reduce inflammation in vivo in a peritonitis model [29,30]. In particular, bone marrow-MSCs injected into the abdominal cavity form aggregates with host macrophages, B cells, and T cells to reduce inflammation in vivo [29,31]. Gosemann et al. reported that hAFSCs injected into the abdominal cavity regulated the acquired immune response via T cells and improved distant organ damage using an LPS-induced peritonitis model [32]. However, the efficacy of hAFSC administration in other animal models of peritonitis and the relationship between hAFSCs and acquired immune responses via T cells, especially regulatory T cells (Treg), has not been completely elucidated.

Hence, in this study, we aimed to investigate the anti-inflammatory effects of hAFSCs in a peritonitis model induced by TG and determine the effects of hAFSCs on in vivo acquired immunity mainly regulated by T cells. We found that hAFSCs ameliorated the TG-induced inflammation by forming aggregates with host immune cells in the peritoneal cavity. Further, hAFSC administration increased the Treg cells in the peritoneal cavity.

## 2. Results

### 2.1. Isolation and Characterization of hAFSCs

**Figure 1:**
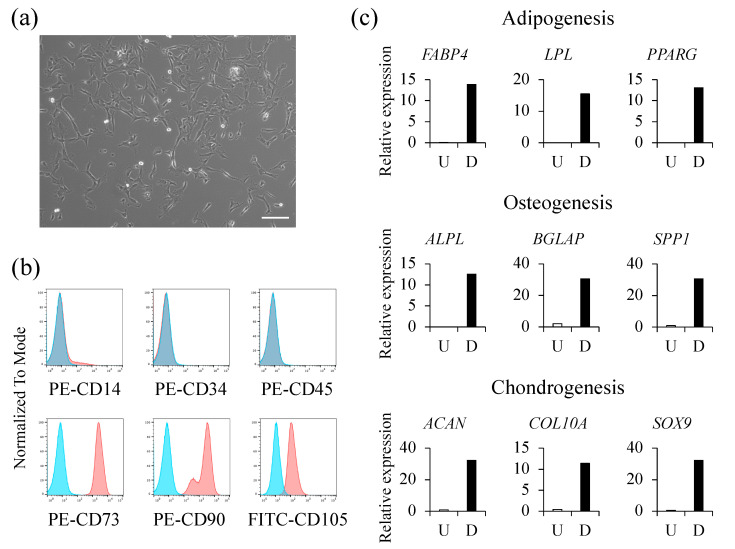
Characteristics of human amniotic fluid stem cells (hAFSCs). (**a**) A bright-field image shows the morphology of hAFSCs in culture (scale bar = 200 µm); (**b**) Surface CD markers of hAFSCs were analyzed by flow cytometry; (**c**) hAFSCs were cultured using adipogenic, osteogenic, or chondrogenic differentiation media. To assess the differentiation potential, the expression of genes characteristic of each differentiated cell was assessed using RT-qPCR. U, undifferentiated; D, differentiated.

The hAFSCs were evaluated for their differentiation potential and surface markers to ensure that they met the definition of MSCs. As reported in our previous studies [18], we expanded the amniotic fluid-derived stem cells isolated by the CD117 magnetic sorting kit using cultures in a growth medium, as described in our previous studies [16,17] (Figure 1a). The hAFSCs did not express hematopoietic surface markers (CD14, CD34, and CD45) but expressed mesenchymal markers (CD73, CD90, and CD105) (Figure 1b). Further, they exhibited the ability to differentiate into adipocytes, osteocytes, and chondrocytes (Figure 1c). 

### 2.2. Treatment with hAFSCs Modulates Peritoneal Inflammation

**Figure 2:**
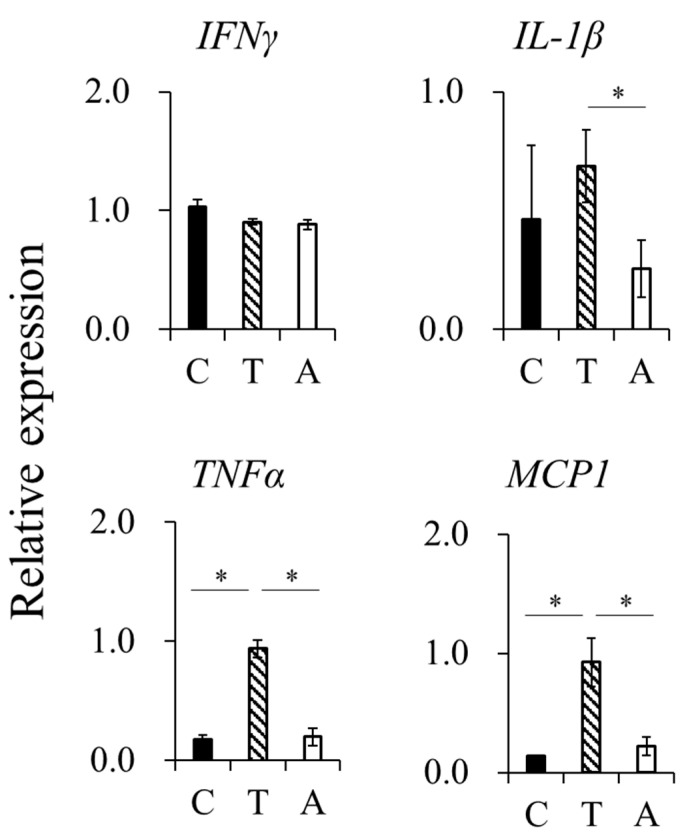
Levels of the pro-inflammatory cytokines. mRNA expressions of interferon gamma (IFNγ), interleukin 1 beta (IL-1β), tumor necrosis factor alpha (TNFα), and monocyte chemoattractant protein-1 (MCP-1) were investigated using RT-qPCR (*n* = 5) in lavaged cells in control (C), thioglycollate (TG) (T), and TG + hAFSCs (A) groups. Results are presented as mean ± SEM. Statistical differences between groups were assessed using analysis of variance and Tukey’s honest significant difference. * *p* < 0.05.

Injection of TG into the peritoneal cavity induces inflammation. Therefore, we examined the mRNA expression levels of genes encoding the pro-inflammatory cytokines interferon gamma (IFNγ), interleukin 1 beta (IL-1β), tumor necrosis factor alpha (TNFα), and monocyte chemoattractant protein-1 (MCP-1) in intraperitoneal washout cells 48 h after TG administration. Their expression increased in the group that received only TG, but decreased in the group that had received hAFSCs before TG administration (Figure 2). 

### 2.3. Engrafted hAFSCs form Aggregates in the Peritoneal Cavity

**Figure 3:**
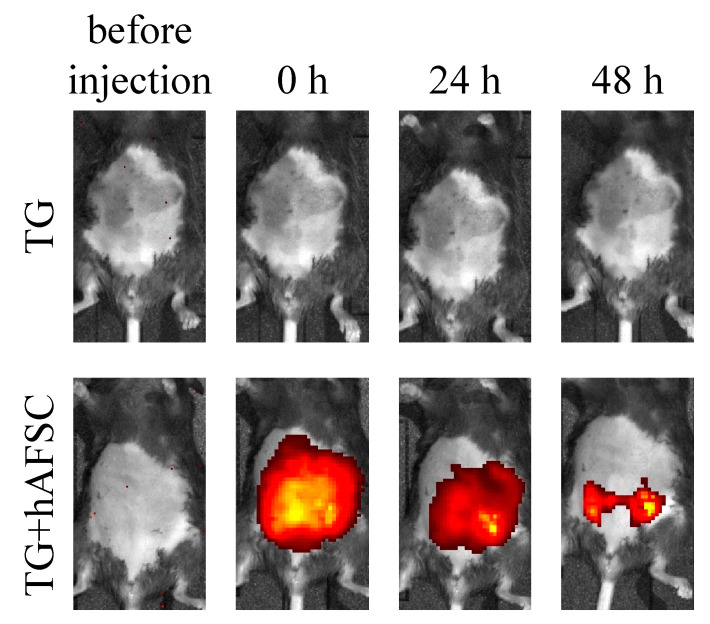
Distribution of DiR-labelled hAFSCs after intraperitoneal implantation. Distribution of hAFSCs (*n* = 5) after injection was determined using in vivo imaging (IVIS^®^). Images display the ventral side and reveal the aggregation of transplanted cells over time.

**Figure 4:**
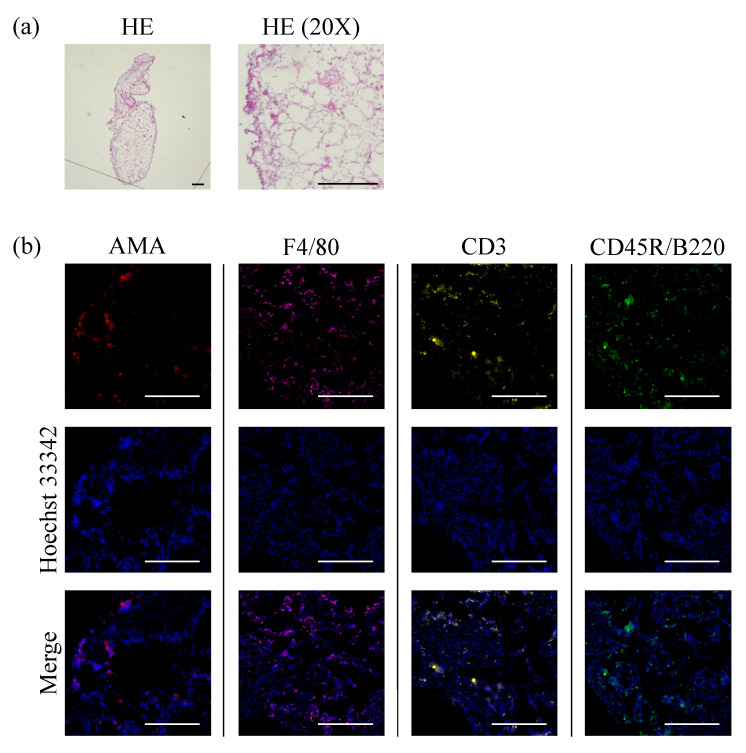
Microscopic analysis showing cellular aggregates. The aggregates were composed of immune cells (macrophages, T cells, and B cells) and human mitochondria-positive hAFSCs (*n* = 5). (**a**) Representatives image of the aggregates obtained by staining with hematoxylin & eosin (HE) stain (Scale bar = 100 µm) (**b**) The aggregates contained anti-human mitochondrial antibody (AMA)-positive hAFSCs. They were also composed of F4/80^+^ peritoneal macrophages, CD3^+^ T cells, and CD45R/B220^+^ B cells (scale bar = 200 µm).

To investigate the localization of hAFSCs in the abdominal cavity, we labeled the hAFSCs with a highly biocompatible fluorescent dye. We examined the localization of hAFSCs using an in vivo imaging system (IVIS^®^) before, immediately after, and 24 and 48 h after hAFSC administration. The fluorescence emitted by the cells gradually aggregated (Figure 3), and cell aggregates were observed in the mice’s abdominal cavity after 48 h (Figure 4a). Certain regions of the cell aggregates were positive for anti-human mitochondria-specific antibodies, macrophage marker F4/80 antibodies, T cell marker CD3, and B cell marker CD45R/B220 (Figure 4b). These results indicated that the intraperitoneally transplanted hAFSCs aggregated with host immune cells.

### 2.4. Administration of hAFSCs Increases the Number of Regulatory T Cells in the Abdominal Cavity

**Figure 5:**
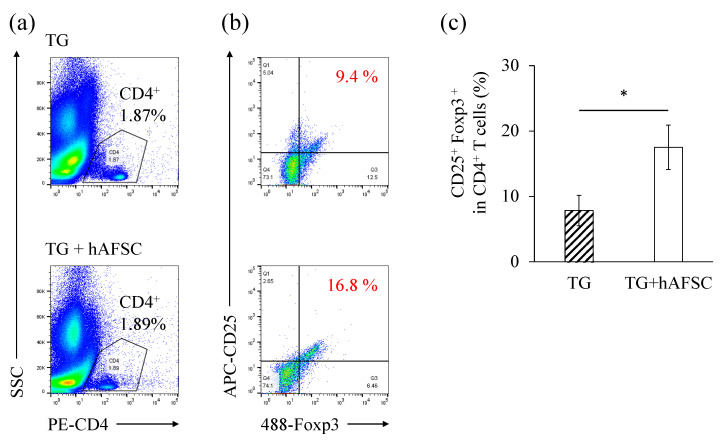
Regulatory T cell (Treg) analysis of the lavaged cells. Ratio of CD4^+^ CD25^+^ Foxp3^+^ Tregs. (**a**, **b**) The gating strategy used is shown. (**c**) The proportion of CD4^+^ CD25^+^ Foxp3^+^ Tregs is shown as a graph (*n* = 5). The data are shown as the means ± SEM. Significance was determined by the Student’s *t* test. * *p* < 0.05.

hAFSCs switch the polarity of macrophages in the abdominal cavity from the inflammatory type (M1) to the anti-inflammatory type (M2) [18]. However, there are immune cells other than macrophages in the abdominal cavity, especially those involved in acquired immunity. One such immune cell of the acquired immune system is the Treg. To detect Tregs, CD4^+^ cells were gated from a total cell population (Figure 5a), and CD25^+^ and Foxp3^+^ populations were further gated (Figure 5b). The ratio of the CD4^+^ CD25^+^ Foxp3^+^ population (Treg) was significantly higher in the hAFSC-treated groups compared with the TG-treated group (Figure 5c). The number of intraperitoneal lavaged cells in mice not receiving TG was so low that it could not be evaluated.

## 3. Discussion

In this study, we found that hAFSC therapy significantly modulated the inflammation induced by intraperitoneal-TG injection in a mouse model of peritonitis. Our in vivo experiments demonstrated that hAFSC administration significantly modulated TG-triggered activation of pro-inflammatory cytokines in the abdominal cavity. hAFSCs formed aggregates with host immune cells, such as T cells, B cells, and macrophages in the peritoneal cavity and survived for at least 48 h. Furthermore, hAFSC treatment increased the number of Treg cells in the peritoneal cavity. To our knowledge, this is the first in vivo study to demonstrate that hAFSC administration increases Treg levels in rodent models of peritonitis.

Further, we investigated the relationship between hAFSCs and T cells. hAFSCs affect immune cells and are highly anti-inflammatory [13,14,15,17,18,33,34,35]. Administration of hAFSCs into the peritoneal cavity is a safe procedure and allows their migration, homing, and integration into various organs of healthy—and inflammation-induced—newborn rats. Moreover, hAFSCs have strong immunomodulatory activity via innate immunity, mainly through macrophages, in neonatal rats [18,33]. Unfortunately, since innate immunity and not acquired immunity plays a central role in neonates, we did not elucidate the effect of hAFSCs on T cells using neonatal rat models in our previous studies. Hence, to investigate the effects of hAFSCs on cells associated with acquired immunity, we focused on the relationship between transplanted hAFSCs and T cells in the host’s abdominal cavity.

Injection of TG into the peritoneal cavity is a traditional method of inducing inflammation in the peritoneal cavity and promoting the migration of inflammatory cells, including T cells [36]. In the abdominal cavity, there are immune cells, such as macrophages and lymphocytes, and the number of these cells increases in response to various stimuli [37,38]. Compared to the LPS-induced peritonitis model in mice, we obtained more lavaged immune cells, such as macrophages and lymphocytes, in the TG-treated model (data not shown). 

Our in vivo experiments showed that the TG-administered hAFSCs aggregated and modulated the inflammation induced by TG in the abdominal cavity. A previous hAFSC-tracking study demonstrated that hAFSCs formed aggregates with host immune cells in the abdominal cavity within 48 h, suggesting that these aggregates could contribute to the modulation of inflammation. Previous reports of the therapeutic effects of hAFSCs in neonatal sepsis rats showed that they aggregated mainly with macrophages [14,18]. However, in the present study, we found that hAFSCs injected into the abdominal cavity of adult rodents accumulated and also aggregated with T cells and B cells in addition to macrophages. These findings were consistent with a previous report on bone marrow-MSC treatment in a mouse model of ulcerative colitis [31]. Thus, hAFSCs form aggregates with the host immune cells critical for regulating innate and acquired immunity, resulting in the modulation of inflammation in the abdominal cavity.

We found that the intraperitoneal administration of hAFSCs increased the number of Tregs in vivo (Figure 5). Tregs (CD4^+^ CD25^+^ Foxp3^+^) suppress immune responses to infectious pathogens, cancer, allogeneic organs, and stem cell transplantation [39,40,41]. The induction of Tregs by MSCs is widely known. The proposed mechanisms include the secretion of soluble mediators by MSCs, cell-to-cell contact, and the regulation of antigen-presenting cells [6,42,43]. In vivo and in vitro studies have shown that MSC therapy increases the number of Tregs in the host [44,45,46]. Unfortunately, there is limited evidence on the effect of hAFSCs on Tregs. In particular, the relationship between hAFSCs and Tregs in vivo has not been investigated, although an in vitro study showed that hAFSCs increased Tregs [47]. The present study fills this gap as it shows that hAFSCs also increase Tregs in vivo. The ability of Tregs to regulate immune regulation in vivo can be present in other MSCs as well, such as bone marrow- and adipose-derived MSCs. 

However, this study has some limitations. First, the composition of the intraperitoneal wash cells needs to be comprehensively investigated. With the advent of flow cytometry, multiplex staining of cells has become easier. However, flow cytometry of intraperitoneal lavaged cells from hAFSC-treated mice is complex. This is because the administered hAFSCs and host macrophages express several cell-adhesion factors [43] that cause many cells to become attracted to each other and become doublets. In our experiments using flow cytometry (hAFSC and lavaged cells), we also observed the following events: human cell-specific antibody positive, mouse macrophage antibody positive, and mouse T cell antibody positive (data not shown). This indicates a direct contact between the administered hAFSCs, host macrophages, and T cells. Thus, it is necessary to disengage these cells without damaging them prior to flow cytometric analysis since clarifying the cell composition in the peritoneal cavity will help unravel the effects of hAFSCs on immune cells in more detail. 

Second, a detailed analysis of the expressions of anti-inflammatory cytokines and their downstream pathways is needed. It needs to be clarified whether these genes are expressed in transplanted cells or in host immune cells. This requires a comprehensive analysis employing detailed sequencing and PCR targeting human- and mouse-specific sequences.

Finally, we have not sufficiently investigated the specific regulatory mechanisms of Tregs. Future studies should elucidate the mechanism of Treg regulation by hAFSCs in more detail. For example, the effects of soluble mediators and cell-to-cell contacts should be investigated through the co-culture of T cells and hAFSCs directly or indirectly using semipermeable membranes. The prevailing hypothesis is that MSCs regulate the Th17/Treg axis through the mTOR pathway. mTOR/HIF-1α-mediated metabolic reprogramming and mTOR/STATs-mediated modification of signaling pathways have been reported to be effective in resolving Th17/Treg imbalances [44]. However, these mechanisms are extremely complex and need to be resolved in the future.

## 4. Materials and Methods

### 4.1. Isolation, Culture, and Characterization of hAFSCs

This study was approved by the institutional review board of the Keio University School of Medicine (No. 20140285). Amniotic fluid collection and culture of hAFSCs were performed in the same way as previously described [14,18]. Briefly, amniotic fluid was obtained from three healthy pregnant women who underwent amniotic fluid examination at 15–16 weeks of gestation and it was cultured in a growth medium. Subsequently, we confirmed that the CD117-positive cells isolated with magnetic beads fulfilled the characteristics of MSCs by analyzing the surface antigen markers and inducing differentiation [18]. 

### 4.2. Animals and TG Medium Injection into the Peritoneal

All experiments were approved by the Animal Committee of Keio University, Japan (No. 21033-(0)). Adult C57BL/6J mice (Charles River Laboratories Japan Inc., Kanagawa, Japan) were bred in an appropriate environment. PBS (2 mL) or TG medium (BD BBLTM Thioglycollate medium Brewer Modified #211716, BD Biosciences, San Jose, CA, USA) (2 mL) was administered intraperitoneally with a 26 G needle (NN-2613S; Terumo, Tokyo, Japan) attached to a syringe (SS-02SZ; Terumo). After 15 min, animals were intraperitoneally injected with either PBS or PBS containing 2 × 10^6^ suspended hAFSCs. After TG administration, the animals were observed every 24 h for two days. 

### 4.3. Collection of Lavage Cell

At 48 h post-TG administration, the animals were anesthetized with 5% isoflurane (Abbott Laboratories, Chicago, IL, USA). To collect the intraperitoneal cells, we injected cold PBS (5 mL) intraperitoneally and gently massaged the abdomen. The intraperitoneal fluid was collected with a syringe as previously reported [17]. The fluid was centrifuged at 500× *g* and 4 °C for 10 min. After discarding the supernatant, the cell pellets were subjected to RNA extraction (see “Real-time qPCR”). 

### 4.4. Tracking hAFSCs after Intraperitoneal Administration and Immunofluorescence Staining of Cell Aggregates

An in vivo imaging system was used to track the administered hAFSCs, and the cell aggregates found in the abdominal cavity were immunostained. The detailed methods are described elsewhere [16,17]. Briefly, XenoLight DiR-labeled hAFSCs were administered intraperitoneally and imaged using IVIS^®^ Spectrum (PerkinElmer, Waltham, MA, USA). We fixed the intraperitoneal cell aggregates with 4% paraformaldehyde and prepared frozen sections (8 µm). These slides were stained with hematoxylin and eosin, anti-human mitochondrial antibody, anti-T cell antibody, anti-B cell antibody and anti-macrophage antibody (see Supplementary Table S1 for antibody information). Stained sections were observed under a confocal laser microscope (LSM710; Zeiss, Jena, Germany).

### 4.5. Flow Cytometry

To investigate the characteristics of intraperitoneal T cells, the lavaged cells were centrifuged from intraperitoneal washings and were analyzed for surface antigens by flow cytometry. A PerFix nc kit (Beckman Coulter Inc., Brea, CA, USA) was used and the manufacturer’s instructions were followed. Briefly, 50 µL of lavaged cells were fixed with the Fixative Reagent and incubated for 15 min at room temperature. Subsequently, 300 µL of Permeabilizing Reagent was added and stained by the antibodies or isotype controls custom mixture for 60 min. The mixture was incubated with PE-conjugated antibodies against CD4, Alexa Fluor 488-conjugated antibodies against Foxp3, APC-conjugated antibodies against CD25, and appropriate isotype controls (Supplementary Table S2). Finally, the sample was washed once with the final solution. Stained cells were analyzed using a MoFlo Astrios EQs cell sorter (Beckman Coulter) and data analyses were performed using FlowJo v10 software (TreeStar Inc., Ashland, OR, USA).

### 4.6. Real-Time qPCR

Total RNA was isolated from the cells using the RNeasy Mini Kit (Qiagen, Hilden, Germany), following the manufacturer’s protocol. Reverse transcription to cDNA was performed using the Prime Script RT Master Mix (Takara Bio, Shiga, Japan). Quantitative PCR (25 µL reaction volume) was performed in duplicate using a 96-well Bio-Rad CFX96 Real-time PCR System (Bio-Rad, Inc., Hercules, CA, USA). The thermocycling conditions were as follows: 50 cycles of 95 °C for 30 s, 95 °C for 5 s, and 60 °C for 20 s. Relative expression per sample was calculated using the 2^−ΔΔCT^ method and was normalized to *GAPDH* expression. The primers used are listed in Supplementary Table S3.

### 4.7. Statistical Analysis

Results are presented as the mean ± standard error of the mean of at least five (*n* = 5) independent experiments. Statistically significant differences between groups were assessed using the *t*-test or analysis of variance and Tukey’s honest significant difference, whichever was appropriate. All statistical analyses were performed using IBM SPSS software (version 25, IBM Corporation, Armonk, NY, USA). Statistical significance was set at *p* < 0.05. 

## 5. Conclusions

This is the first study to demonstrate the effects of hAFSCs on Tregs in vivo using a rodent model of peritonitis induced by TG injection into the abdominal cavity. We showed that hAFSCs ameliorated the TG-induced inflammation, probably by forming aggregates with host immune cells such as macrophages, T cells, and B cells in the peritoneal cavity. These results indicated that hAFSCs could activate both the innate and acquired immunity in vivo against inflammation-related diseases.

## Data Availability

The data presented in this study are available on request from the corresponding author.

## Supplementary Materials

The following supporting information can be downloaded at: https://www.mdpi.com/article/10.3390/ijms23126433/s1.
