# Supplementary material for: Human Amniotic Fluid Stem Cells Ameliorate Thioglycollate-Induced Peritonitis by Increasing Tregs in Mice
Source: Int J Mol Sci. 2022 Jun 9;23(12):6433. doi: 10.3390/ijms23126433 (PMC9224120; doi:10.3390/ijms23126433)
Supplement: Supplementary file 1 [file ijms-23-06433-s001.zip › ijms-1670508-supplementary.pdf]

Table S1. List of antibodies used for immunohistochemistry.

**Antigen**	**Host**	**Type**	**Dilution**	**Code**	**Source**
Human Mitochondria	Mouse	Monoclonal	1:50	MAB1273C3	Sigma-Aldrich
F4/80	Rat	Monoclonal	1:500	MCA497RT	Bio-Rad
CD3	Rabbit	Polyclonal	1:100	ab5690	Abcam
CD45R/B220	Rat	Monoclonal	1:100	103239	BioLegend

Table S2. List of antibodies used for flow cytometry in the present study.

Species	Antigen	Clone	Fluorochrome	Manufacture
CD14	Human	HCD14	PE	BioLegend
CD34	Human	581	PE	BioLegend
CD45	Human	HI30	PE	BioLegend
CD73	Human	AD2	PE	BioLegend
CD90	Human	5E10	PE	BioLegend
CD105	Human	43A3	FITC	BioLegend
CD4	Mouse	RM4-5	PE	BioLegend
CD25	Mouse	PC61	APC	BioLegend
FOXP3	Mouse	MF-14	Alexa Fluor 488	BioLegend

Table S3. List of primer sequences used for RT-qPCR in the present study.

Gene	Forward primer (5′-3′)	Reverse primer (5′-3′)
**Human**		
GAPDH	TCAAGGCTGAGAACGGGAAG	CGCCCCACTTGATTTTGGAG
FABP4	AACCTTAGATGGGGGTGTCC	ATGCGAACTTCAGTCCAGG
LPL	GAGATTTCTCTGTATGGCACC	CTGCAAATGAGACACTTTCTC
PPARG	GCTGTTATGGGTGAAACTCTG	ATAAGGTGGAGATGCAGGC
ALPL	CTGGTAGGCGATGTCCTTA	ACGTGGCTAAGAATGTCATC
BGLAP	CTCACACTCCTCGCCCTA	CCTGAAAGCCGATGTGGT
SPP1	ATCACCTGTGCCATACCAGT	CACATCGGAATGCTCATTGC
ACAN	GATGTTCCCTGCAATTACCACCTC	TGATCTCATACCGGTCCTTCTTCTG
COL10A1	CATAAAAGGCCCACTACCCAAC	ACCTTGCTCTCCTCTTACTGC
SOX9	GGAGATGAAATCTGTTCTGGGAATG	TTGAAGGTTAACTGCTGGTGTTCTG
**Mouse**		
GAPDH	CCTGCTTATCCAGTCCTAGCTC	GCTTGGTGCGTGCACATT
IFNγ	AGCAAGGCGAAAAAGGATGC	TCATTGAATGCTTGGCGCTG
IL-1β	TGCCACCTTTTGACAGTGATG	TGATGTGCTGCTGCGAGATT
TNFα	CTGTAGCCCACGTCGTAGC	TTGAGATCCATGCCGTTG
MCP-1	TCCCACTCACCTGCTGCTACTCA	GCTTCTTTGGGACACCTGCTG
